# Montelukast, a Cysteinyl Leukotriene Receptor 1 Antagonist, Induces M2 Macrophage Polarization and Inhibits Murine Aortic Aneurysm Formation

**DOI:** 10.1155/2019/9104680

**Published:** 2019-05-27

**Authors:** Yohei Kawai, Yuji Narita, Aika Yamawaki-Ogata, Akihiko Usui, Kimihiro Komori

**Affiliations:** ^1^Division of Vascular Surgery, Department of Surgery, Nagoya University Graduate School of Medicine, Nagoya, Japan; ^2^Department of Cardiac Surgery, Nagoya University Graduate School of Medicine, Nagoya, Japan

## Abstract

**Background:**

The pathogenesis of abdominal aortic aneurysm (AAA) is characterized by atherosclerosis with chronic inflammation in the aortic wall. Montelukast is a selective cys-LT 1 receptor antagonist that can suppress atherosclerotic diseases. We evaluated the in vitro properties of montelukast and its in vivo activities in an angiotensin II–infused apolipoprotein E–deficient (apoE^−/−^) AAA mouse model.

**Methods:**

The mouse monocyte/macrophage cell line J774A.1 was used in vitro. M1 macrophages were treated with montelukast, and gene expressions of inflammatory cytokines were measured. Macrophages were cultured with montelukast, then gene expressions of arginase-1 and IL (interleukin)-10 were assessed by quantitative polymerase chain reaction, arginase-1 was measured by fluorescence-activated cell sorting, and IL-10 concentration was analyzed by enzyme-linked immunosorbent assay. In vivo, one group (Mont, n=7) received oral montelukast (10 mg/kg/day) for 28 days, and the other group (Saline, n=7) was given normal Saline as a control for the same period. Aortic diameters, activities of matrix metalloproteinases (MMPs), cytokine concentrations, and the number of M2 macrophages were analyzed.

**Results:**

Relative to control, montelukast significantly suppressed gene expressions of MMP-2, MMP-9, and IL-1*β*, induced gene expressions of arginase-1 and IL-10, enhanced the expression of the arginase-1 cell surface protein, and increased the protein concentration of IL-10. In vivo, montelukast significantly decreased aortic expansion (Saline vs Mont; 2.44 ± 0.15 mm vs 1.59 ± 0.20 mm, P<.01), reduced MMP-2 activity (Saline vs Mont; 1240 *μ*M vs 755 *μ*M, P<.05), and induced infiltration of M2 macrophages (Saline vs Mont; 7.51 % vs 14.7 %, P<.05).

**Conclusion:**

Montelukast induces M2 macrophage polarization and prevents AAA formation in apoE^−/−^ mice.

## 1. Introduction

Abdominal aortic aneurysm (AAA) is a localized dilatation of the aorta and is a life-threatening vascular disorder [1]. The usual treatment for AAA is open surgical repair or endovascular repair to prevent rupture of the aorta. Although these techniques are effective in patients with a large AAA, few therapeutic strategies are available for surgically unsuitable patients who have a small AAA or who are otherwise at high risk or cannot undergo open or endovascular surgery because of anatomical limitations. Therefore, nonsurgical or less invasive therapeutic procedures for the treatment of AAA are urgently required. The purpose of our study was to verify whether or not montelukast, a cys-LT 1 receptor antagonist, would be effective as a minimally invasive treatment, and how macrophages were involved in its mechanism of action.

The pathogenesis of AAA is characterized by the destruction of the extracellular matrix, with chronic inflammation in the aortic wall. This inflammation is induced by the infiltration and activation of inflammatory cells such as macrophages, which release inflammatory cytokines and matrix metalloproteinases (MMPs) that cause degradation of the extracellular matrix [2].

Macrophages play an important role in regulating the inflammatory process in AAA. The two main macrophage phenotypes are M1 and M2 [3]. M1 macrophages are release proinflammatory cytokines and strongly promote aneurysm formation. In contrast, M2 macrophages are considered to be anti-inflammatory and are highly protective against elastin degradation and aneurysm progression. Therefore, the M1/M2 macrophage ratio plays a critical role in aneurysm development and is potentially a significant therapeutic target [4, 5].

Leukotrienes (LTs), which are derived from the 5-lipoxygenase (5-LO) pathway of arachidonic acid metabolism, are potent lipid mediators with proinflammatory biological actions [6]. Among them, cysteinyl-leukotrienes (cys-LTs), which include LTC_4_, LTD_4_, and LTE_4_, increase vascular permeability and smooth muscle cell contraction, leading to bronchoconstriction and vasoconstriction in asthmatic patients [7]. Recent experimental studies reported that LTD_4_ induced MMP production through a cys-LT 1 receptor in the aortic wall [8] and that montelukast, a selective cys-LT 1 receptor antagonist with anti-inflammatory effects that has been widely used for the treatment of inflammatory diseases such as asthma and allergic rhinitis, inhibited the development of atherosclerotic diseases [9, 10]. Recently, Gennaro et al. showed that montelukast prevented experimental AAA [11]. However, little information is available about the role of montelukast in AAA formation. Therefore, we evaluated the in vitro properties of montelukast and its in vivo activities in an angiotensin II (Ang II)–induced apolipoprotein E–deficient (apoE^−/−^) AAA mouse model.

## 2. Materials and Methods

This study was performed in accordance with the Guide for the Care and Use of Laboratory Animals (National Institutes of Health, Publication 85-23, National Academy Press, Washington, DC, USA, revised in 2011) and was approved by the Institute for Laboratory Animal Research at the Nagoya University Graduate School of Medicine (Protocol No. 30003).

### 2.1. Cell Culture and Treatment

Murine macrophages of the J774A.1 cell line were purchased from the National Institutes of Biomedical Innovation, Health, and Nutrition (Ibaraki, Osaka, Japan) and cultured in Dulbecco's modified essential medium (DMEM) (Gibco, Thermo Fisher Scientific, Waltham, MA, USA) supplemented with 10% fetal bovine serum (FBS; Gibco, Thermo Fisher Scientific). Macrophages were seeded on 24-well tissue culture plates (Iwaki; AGC, Inc., Tokyo, Japan) at a concentration of 4×10^5^ cells per well and incubated for 4 days at 37°C in a humidified atmosphere with 5% CO_2_. To increase the release of MMPs and inflammatory cytokines from inflammatory M1 macrophages, the cells were stimulated by TNF-*α* (20 ng/ml) at 37°C in 5% CO_2_ for 24 hours. After that, the medium was replaced with 1 ml DMEM with 10% FBS containing 2 *μ*M (n=6) or 20 *μ*M (n=6) montelukast (Tokyo Kasei Kogyo Co., Ltd., Tokyo, Japan) for 12 hours [12]. We used the 20 *μ*M dose because a previous report demonstrated that 20 *μ*M montelukast reduced oxidized low-density lipoprotein-induced monocyte adhesion to human umbilical vein endothelial cells [13]. Control wells contained 1 ml of montelukast-free DMEM (n=6).

### 2.2. RNA Isolation and Quantitative Real-Time Polymerase Chain Reaction (qRT-PCR)

Total cellular RNA was extracted from cultured cells with the NucleoSpin RNA kit (Takara Bio Inc., Shiga, Japan) according to the manufacturer's protocol. Samples were normalized to 0.5 *μ*g of total RNA. Then, cDNA was synthesized using the Takara PrimeScript RT reagent kit (Takara Bio Inc.). qRT-PCR was performed using the KAPA SYBR FAST qPCR kit (Kapa Biosystems, Boston, MA, USA). The PCR cycling conditions were as follows: 95°C for 1 minute and then 40 cycles of 15 seconds at 95°C for denaturation, followed by 60°C for 45 seconds for annealing. To amplify macrophage genes, we selected primers for MMP-2, MMP-9, interleukin (IL)-1*β*, IL-6, monocyte chemotactic protein (MCP)-1, TNF-*α*, IL-10, and arginase-1, along with glyceraldehyde 3-phosphate dehydrogenase (GAPDH) as a control (Table 1) [12].

### 2.3. Flow Cytometry

Murine macrophages of the J774A.1 cell line were cultured with (n=3) or without (n=3) 20 *μ*M montelukast for 96 hours. To analyze immunophenotyping of a specific cell surface antigens of anti-inflammatory M2 macrophages, flow cytometry was performed. Markers of arginase-1 (arginase-1 monoclonal antibody–PE, eBioscience, 1:100; Invitrogen, Thermo Fisher Scientific) and of mouse IgG2s K isotype as a negative control (1:100; Invitrogen, Thermo Fisher Scientific) were measured on montelukast-treated and control macrophages using a FACS Calibur (Becton Dickinson, NJ, USA) and FlowJo software ver 9.9.4 (FlowJo LLC, Oregon, USA) [4].

### 2.4. Animal Preparation and Drug Administration

AAA model mice were generated as previously described [14, 15]. Briefly, apoE^−/−^ mice on the C57BL/6 background were purchased from the Jackson Laboratory (Sacramento, CA, USA) and maintained on a regular diet under standard conditions (24°C, 63% humidity). Male apoE^−/−^ mice (6 to 8 months old) were infused with 1000 ng/kg/min Ang II (Calbiochem, Darmstadt, Germany) for 28 days through subcutaneous osmotic minipumps (model 2004; DURECT, Cupertino, CA, USA) that were implanted under anesthesia with isoflurane. Several studies reported that the efficacy of montelukast against asthma was dose dependent when administered orally [16–18], so we presumed that its effectiveness in this setting could be concentration dependent. Therefore, one group (Mont, n=7) received oral montelukast (10 mg/kg/day) through a gastric tube once a day for 28 days, and the other group (Saline, n=7) was administered oral normal Saline for the same period (Supplementary Figure 1). We examined the maximum AAA diameters of mice by ultrasonography (10 MHz; GE Healthcare, Chicago, IL, USA) before pump implantation and every week thereafter. Mice were sacrificed at 28 days after administration, and the portion of the aorta between the descending aorta and the iliac bifurcation was harvested. The maximum AAA diameter from the thoracoabdominal aorta to the suprarenal abdominal aorta was measured, as previously described [14, 15]. AAA was defined as a 50% increase relative to the external aortic diameter before pump implantation [19]. Images of aortas were captured with a DP70 digital camera (Olympus, Tokyo, Japan) connected to stereomicroscope, and 2-mm-long segments of isolated aorta were cut at the infradiaphragm for histologic examination. The residual aorta was minced and homogenized for analysis of MMP enzymatic activity and protein expression.

### 2.5. Elastica Van Gieson Staining

For the determination of elastin degradation, we performed Elastica van Gieson staining as previously described [14]. Briefly, harvested aortas were embedded in OCT compound (Tissue-Tek; Sakura Finetek USA, Torrance, CA, USA), and we prepared frozen slices (8-*μ*m thickness) using a microtome cryostat (Leica microsystems). The frozen cross-sections were stained with Weigert's resorcin-fuchsin (Muto Pure Chemicals, Tokyo, Japan) according to the manufacturer's protocol, and stained samples were photographed with an FSX-100 microscope (Olympus, Tokyo, Japan). The number of intact elastin fibrils on the aortic wall was analyzed using ImageJ software (National Institutes of Health, Bethesda, MD, USA). The images were used to quantify the percent area of elastin and the percent area of medial components between the elastic lamellae relative to the total medial tissue area [20].

### 2.6. MMP-2 and MMP-9 Enzymatic Activity

To assay MMP activity, aortic tissues were homogenized with protein extraction buffer containing 20 mM Tris-HCl (pH 7.6), 150 mM NaCl, 50 mM CaCl_2_, 0.01% Brij-35 (MP Biomedicals, Santa Ana, CA, USA), and 1% Triton-X 100. MMP-2 activity in aortic tissues was determined using the MMP-2 activity assay kit (SensoLyte 520; AnaSpec, San Jose, CA, USA), and activity of MMP-9 was detected using the MMP-9 activity assay kit (QuickZyme BioSciences, Netherlands) according to the manufacturer's protocol [21].

### 2.7. Enzyme-Linked Immunosorbent Assay (ELISA)

Aortic tissues and 20 *μ*M montelukast-induced macrophages were homogenized using an ultrasonic disintegrator (Sonics & Materials, Inc., Newtown, CT, USA) in protein extraction buffer (CytoBuster; Novagen, Merck KGaA, Darmstadt, Germany) with 20 mM of ethylenediaminetetraacetic acid (Dojindo, Kumamoto, Japan) and 1 mM of phenylmethylsulfonyl fluoride (Thermo Fisher Scientific). Lysate protein concentration was measured using the Qubit® Protein Assay Kit and Qubit® 2.0 fluorometer (Thermo Fisher Scientific). An equal concentration of total protein was applied to each assay kit and detected according to the manufacturer's protocol for each ELISA kit (TIMP-1 and TGF-*β*1: R&D Systems, Minneapolis, MN, USA; TIMP-2: RayBiotech, Norcross, GA, USA; IGF-1: Mediagnost, Reutlingen, Germany; IL-1*β*, IL-6, TNF-*α*, and MCP-1: Bender MedSystems, Vienna, Austria; IL-10: Thermo Fisher, Waltham, MA, USA) [12]. Seven samples were analyzed per group.

### 2.8. Immunofluorescence of M1 and M2 Macrophages

To identify M1 inflammatory and M2 anti-inflammatory macrophages, immunofluorescence was performed as previously described [14, 15]. After frozen sections were fixed and blocked, rabbit polyclonal anti–inducible nitric oxide synthase (iNOS) antibody (1:100; Abcam, Cambridge, UK) and rabbit anti-arginase-1 antibody (1:50; Cell Signaling Technology, Danvers, MA, USA) were used as primary antibodies for staining M1 and M2 macrophages, respectively. Anti-rabbit IgG (H+L) Alexa Fluor 546–conjugated antibody (1:1000; Cell Signaling Technology) and anti-rabbit immunoglobulin G (IgG; H+L) Alexa Fluor 488–conjugated antibody (1:1000; Cell Signaling Technology) were used as secondary antibodies against iNOS and arginase-1 primary antibodies, respectively. Nucleated cells were stained using 4',6-diamidino-2-phenylindole Fluoromount-G (Southern Biotech, Birmingham, AL, USA). Slides were imaged using an Olympus IX51 microscope and DP2-BSW software (Olympus, Tokyo, Japan), and images were analyzed by ImageJ software. For quantification, seven random, nonoverlapping regions were imaged for each AA tissue sample. The number of iNOS or arginase-1-positive nucleated cells was then counted for each image. Images that were stained with DAPI only were used as a control, and the percentage of M1 or M2 macrophages was calculated.

### 2.9. Statistical Analysis

Statistical significance was calculated and compared between 2 groups using the *χ*2 test or unpaired t-test as appropriate, and between 3 groups using one-way ANOVA and subsequent Tukey-Kramer multiple comparison. The SPSS software package (SPSS Inc., Chicago, IL, USA) was used in all cases. Data are shown as the mean ± standard error of the mean. P values less than 0.05 were considered statistically significant.

## 3. Results

### 3.1. Montelukast Suppresses Inflammatory Mediators Induced by TNF-*α*

**Figure 1:**
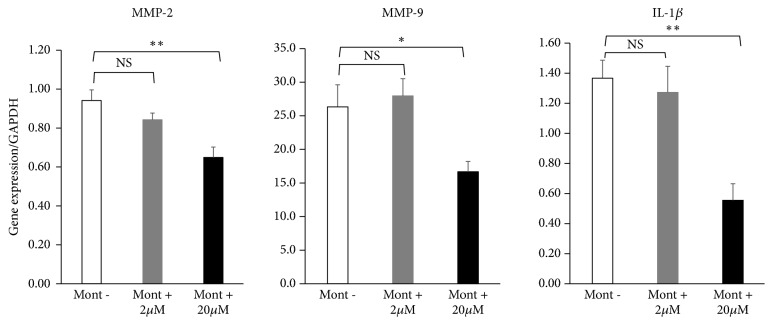
Effects of montelukast on gene expressions of proinflammatory factors in TNF-*α*–stimulated macrophages. These macrophages were incubated for 12 hours in culture medium containing montelukast. Gene expression levels were determined by quantitative RT-PCR and standardized to GAPDH levels. Expressions of MMP-2, MMP-9, IL-1*β*, and NF-*κ*B were significantly lower in macrophages cocultured with 20 *μ*M montelukast (n=6) than in nontreated macrophages (n=6). Data are expressed as the mean ± standard error of the mean and assessed by one-way ANOVA (_ _^*∗*^P < 0.05  _ _^*∗∗*^P < 0.01).

A dose of 20 *μ*M montelukast significantly suppressed macrophages gene expressions of MMP-2 (Mont- vs 20 *μ*M; 0.94 ± 0.06 vs 0.64 ± 0.06, P<.01), MMP-9 (Mont- vs 20 *μ*M; 26.5 ± 3.18 vs 16.7 ± 1.56, P<.05), and IL-1*β* (Mont- vs 20 *μ*M; 1.37 ± 0.12 vs 0.56 ± 0.11, P<.01). However, 2 *μ*M montelukast caused no significant differences compared with control (Figure 1).

### 3.2. Montelukast Induces M2 Macrophages

**Figure 2:**
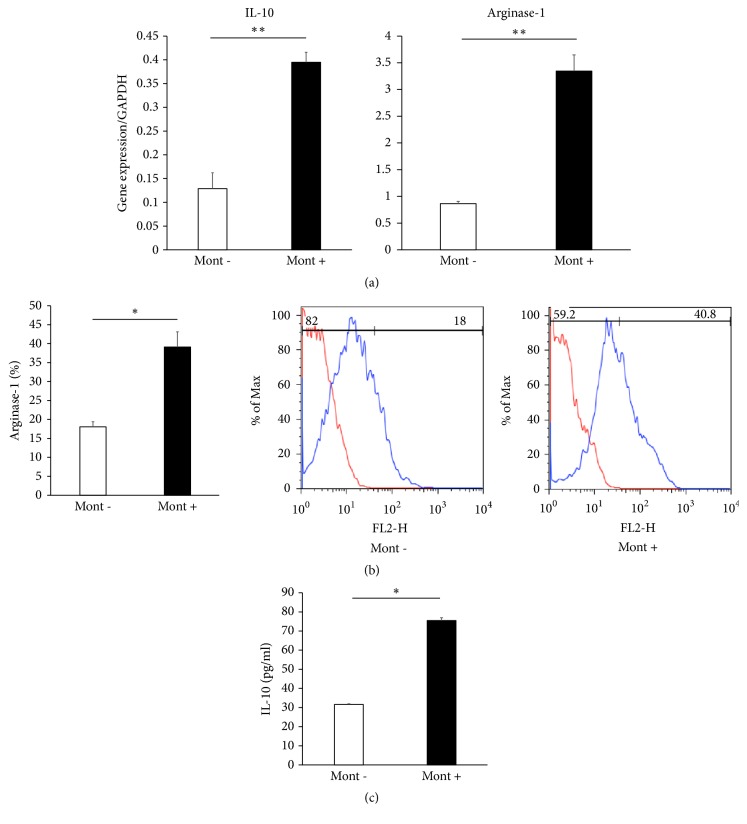
Gene expressions of IL-10 and arginase-1, cell surface antigen expression of arginase-1, and protein concentration of IL-10. Macrophages were incubated for 96 hours with medium with (n=3) or without (n=3) 20 *μ*M montelukast. Gene expressions of IL-10 and arginase-1 were measured by qRT-PCR, arginase-1 cell surface antigen expression on macrophages was analyzed by flow cytometry, and protein concentration of IL-10 was detected by enzyme-linked immunosorbent assay. Macrophages cultured with montelukast demonstrate significantly increased gene expressions of IL-10 and arginase-1 (a), protein expression of arginase-1 (b), and protein concentration of IL-10 (c). Data are presented as the mean ± standard error of the mean. (a) _ _^*∗∗*^P < 0.01, (b, c) _ _^*∗*^P < 0.05.

In macrophages cultured with 20 *μ*M montelukast, qRT-PCR showed significantly increased gene expression of IL-10 (Mont- vs Mont+; 0.13 ± 0.03 vs 0.40 ± 0.02, P<.01) and arginase-1 (Mont- vs Mont+; 0.87 ± 0.05 vs 3.35 ± 0.30, P<.01) (Figure 2(a)), and flow cytometric analysis demonstrated significantly increased expression of arginase-1 (Mont- vs Mont+; 17.8 ± 1.20 % vs 38.5 ± 3.76 %, P<.05, Figure 2(b)). The protein level of IL-10 was significantly higher in macrophages cocultured with 20 *μ*M montelukast than in nontreated macrophages (Mont- vs Mont+; 31.4 ± 0.40 pg/ml vs 75.7 ± 1.29 pg/ml, P<.05, Figure 2(c)).

### 3.3. Aortic Diameter Reduction

**Figure 3:**
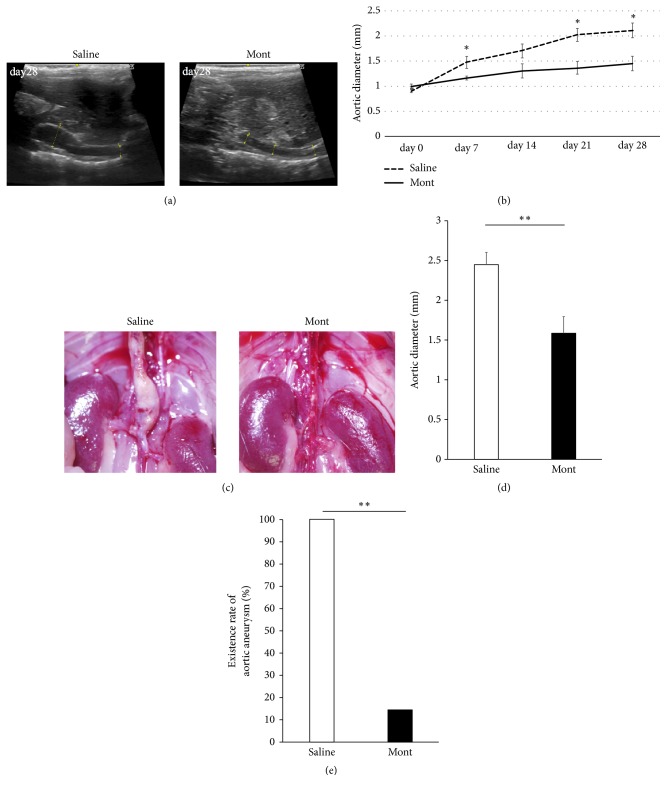
Aortic diameter on ultrasonography, macroscopic findings, aortic diameter, and incidence of aortic aneurysm in the montelukast group (n=7) and the Saline group (n=7). (a) Ultrasonography shows long-axis views of the thoracoabdominal aorta. (b) Aortic aneurysm formation is significantly suppressed in the montelukast group compared with the Saline group. (c) Representative macroscopic images indicate that aortic aneurysms developed in the Saline group but not the montelukast group. (d) At day 28 after implantation, the aortic diameter in the montelukast group is significantly lower than that in the Saline group. (e) The incidence rate of aortic aneurysm is 100% in the Saline group, compared with 14.3% in the montelukast group. Data are presented as the mean ± standard error of the mean. (b) _ _^*∗*^P < 0.05, (d, e) _ _^*∗∗*^P < 0.01.

During this experiment, no mice died or experienced complications. The maximal aortic diameters in the Saline and montelukast groups, respectively, were 0.91 ± 0.04 mm and 0.99 ± 0.06 mm before administration (P=.27), 1.48 ± 0.12 mm and 1.16 ± 0.04 mm at day 7 (P<.05), 1.70 ± 0.14 mm and 1.30 ± 0.14 mm at day 14 (P=.08), 2.02 ± 0.14 mm and 1.36 ± 0.13 mm at day 21 (P<.01), and 2.10 ± 0.15 mm and 1.45 ± 0.15 mm at day 28 (P<.05) (Figures 3(a), 3(b), and 3(c)). At day 28, aortic diameters under microscopy were significantly expanded in the Saline group compared with the montelukast group (Saline vs Mont; 2.44 ± 0.15 mm vs 1.59 ± 0.20 mm, P<.01, Figure 3(d)). The incidence rate of AAAs in the Saline group was significantly higher than that in the montelukast group (Saline vs Mont; 100 % vs 14.3 %, P<.01, Figure 3(e)).

### 3.4. Elastic Lamellae in Medial Layer of the Aortic Wall

**Figure 4:**
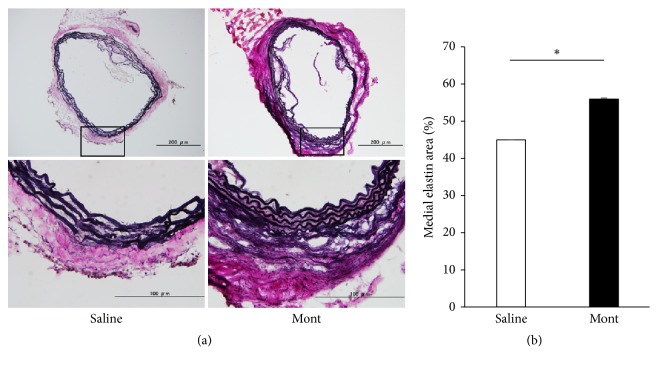
Elastica van Gieson staining. (a) Images show more extensive destruction of the elastic lamellae in the Saline group than in the montelukast group. (b) The medial elastin area is significantly lower in the Saline group (n=7) than in the montelukast group (n=7). Data are presented as the mean ± standard error of the mean. (b) _ _^*∗*^P < 0.05.

The aortic media in the Saline group contained a lower percentage of unbroken elastic lamellae than the montelukast group (Saline vs Mont; 45.0 ± 3.04 vs 56.1 ± 1.62 (%), P<.05) (Figures 4(a) and 4(b)).

### 3.5. Suppression of MMP-2 Activity

**Figure 5:**
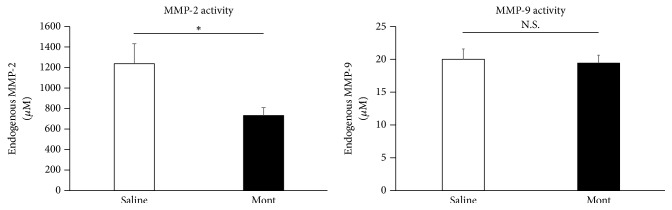
The amounts of MMP-2 and MMP-9 in AAA tissues were calculated. Endogenous MMP-2 is significantly lower in the montelukast group (n=7) than in the Saline group (n=7). Data are presented as the mean ± standard error of the mean (_ _^*∗*^P < 0.05).

The activity of endogenous MMP-2 was significantly lower in the montelukast group than in the Saline group (endogenous MMP-2: Saline vs Mont; 1234 ± 199 *μ*M vs 734 ± 71 *μ*M, P<.05, Figure 5). However, there was no significant difference between the two groups in the activity of endogenous MMP-9.

### 3.6. Expression of Cytokines, Chemokines, and Growth Factors in Aortic Walls

**Figure 6:**
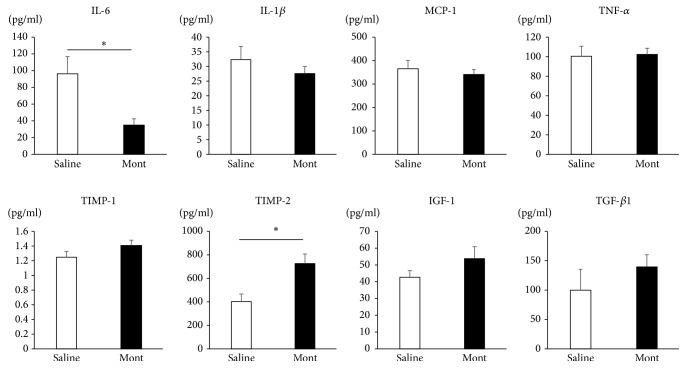
Protein concentration in the aortic tissue, measured by enzyme-linked immunosorbent assay. Compared with the Saline group (n=7), the montelukast group (n=7) demonstrates a significantly lower expression level of IL-6 but a significantly higher level of TIMP-2. Data are presented as the mean ± standard error of the mean (_ _^*∗*^P < 0.05).

There were no significant differences in expression levels of TIMP-2, MCP-1, IL-1*β*, TNF-*α*, IGF-1, or TGF-*β*1 between the Saline and montelukast groups. However, the montelukast group demonstrated a significantly lower expression level of IL-6 (Saline vs Mont; 95.4 ± 21.2 pg/ml vs 34.3 ± 7.70 pg/ml, P<.05) and a significantly higher expression level of TIMP-2 (Saline vs Mont; 400 ± 69.8 pg/ml vs 725 ± 82.6 pg/ml, P<.05) (Figure 6).

### 3.7. Percentages of M1 and M2 Macrophages

**Figure 7:**
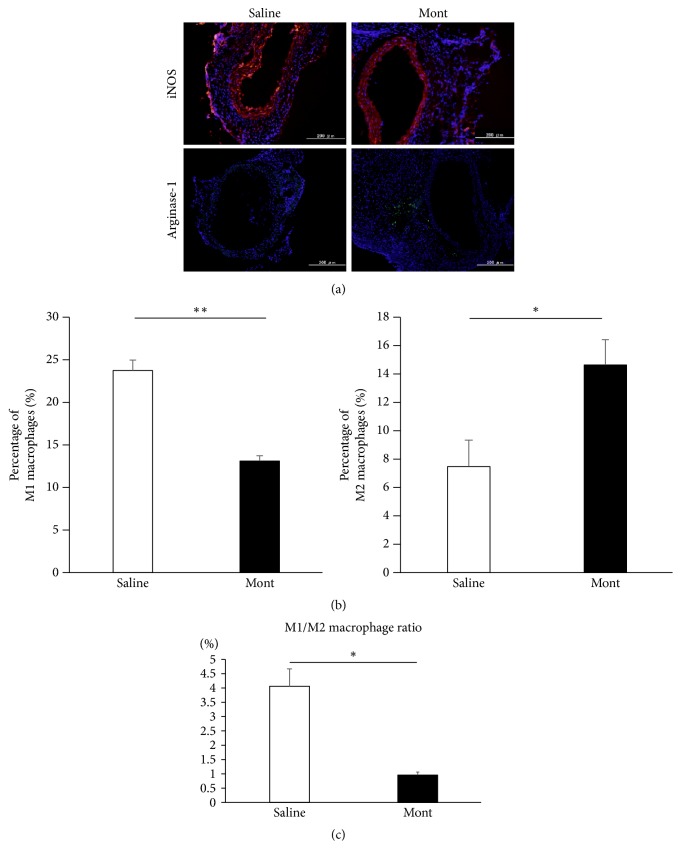
Immunofluorescence staining. (a) In immunofluorescence staining of M1 and M2 macrophages, nitric oxide synthase (iNOS)–labeled M1 macrophages are shown in red, and arginase-1-labeled M2 macrophages are shown in green. Nuclei are stained blue. (b) Quantitative analysis of iNOS-positive and arginase-1-positive cells as ratios relative to the total number of cells (nuclei) shows that the percentage of M1 macrophages is significantly higher in the Saline group than in the montelukast group, and the percentage of M2 macrophages is significantly higher in the montelukast group than in the Saline group. (c) Quantitation of the iNOS-positive cells divided by the arginase-1-positive cells (%). Data are presented as the mean ± standard error of the mean. (b) _ _^*∗*^P < 0.05  and  _ _^*∗∗*^P < 0.01, (c) _ _^*∗*^P < 0.05.

Immunofluorescent staining, shown in Figure 7(a), indicated that the percentage of M1 macrophages (iNOS positive) was significantly higher in the Saline group than in the montelukast group (Saline vs Mont; 23.8 ± 1.20 % vs 13.1 ± 0.72 %, P<.01, Figure 7(b)). In contrast, the percentage of M2 macrophages (arginase-1 positive) was significantly higher in the montelukast group than in the Saline group (Saline vs Mont; 7.51 ± 1.84 % vs 14.7 ± 1.72 %, P<.05, Figure 7(b)). The M1/M2 macrophage ratio was significantly lower in the montelukast group (Saline vs Mont; 8.32 ± 2.34 % vs 0.87 ± 0.25 %, P<.05, Figure 7(c)).

## 4. Discussion

Leukotrienes, which are derived from arachidonic acid by 5-LO in conjunction with 5-LO-activating protein (FLAP), are potent leukocyte chemoattractants and mediators of inflammation. LTA_4_, which is produced from arachidonic acid, is hydrolyzed by LTA_4_ hydrolase into LTB_4_ or conjugated by LTC_4_ synthase (LTC_4_S) to form cys-LTs, including LTC_4_, LTE_4_, and LTD_4_ [9]. Animal and human studies revealed that cys-LTs were involved in the pathogenesis of atherosclerosis and that the 5-LO pathway played an important role in the formation of AAA. Increased expression of cys-LTs was detected in human atherosclerotic lesions [22], and mice with 5-LO gene deletion demonstrated a significant reduction of atherosclerosis [23]. In the walls of human AAA, mRNA levels of 5-LO, FLAP, and LTC_4_S were significantly increased, and incubation of aortic wall tissues with arachidonic acid produced a significant amount of cys-LTs [24]. These findings show that cys-LTs induce the release of MMPs, including MMP-2 and MMP-9, as well as inflammatory cytokines such as ILs and TNF-*α*, from macrophages through cys-LT receptor 1.

Montelukast, a selective cys-LT receptor 1 antagonist, has been widely used to treat asthma and allergic rhinitis. Its mechanisms of action are considered to include stimulating neutrophil infiltration [25], balancing oxidant-antioxidant status, and decreasing plasma C-reactive protein levels. Some studies showed that montelukast could inhibit the inflammatory response related to atherosclerosis [9, 26]. A previous animal study demonstrated that montelukast inhibited the development of atherosclerosis in association with decreased expression of MMP-2 and MMP-9 [27], and another experimental study indicated that montelukast produced anti-atherogenic effects related to MCP-1 downregulation [28]. Recently, Gennaro et al. demonstrated that montelukast reduced experimental AAA by inhibiting the release of MMP-9 and macrophage inflammatory protein-1*α* [11]. However, they did not investigate the relationship between montelukast and anti-inflammatory effects, especially those mediated by M2 macrophages. In contrast, we revealed that M2 macrophage polarization by montelukast participated in the prevention of AAA formation.

We demonstrated that montelukast suppressed the gene expressions of inflammatory cytokines in stimulated macrophages induced by TNF-*α* in vitro. The gene expressions of MMP-2, MMP-9, and IL-1*β* were significantly lower in macrophages cocultured with montelukast than in nontreated macrophages. IL-1*β* is considered to be an important mediator of inflammation and is believed to be critical in experimental AAA formation [29].

While montelukast suppressed the expression of certain genes in our study, it also promoted gene expressions of IL-10 and arginase-1, enhanced the protein expression of arginase-1, and increased the protein concentration of IL-10 in macrophages incubated with montelukast. Our data thus indicate for the first time that montelukast induces M2 macrophages, which play a key role in suppressing AAA formation. Montelukast not only potently inhibits cys-LT but also strongly induces M2 macrophage polarization, and consequently it is reasonable to suppose that administration of montelukast could inhibit the development and expansion of AAA. Moreover, we found that montelukast significantly decreased the infiltration of M1 inflammatory macrophages and increased the infiltration of M2 anti-inflammatory macrophages in an in vivo experiment. The mechanism whereby montelukast prevents AAA formation therefore seems to involve enhancing anti-inflammatory activity by inducing M2 macrophages, leading to a reduction in size of AAA. M2 macrophages are divided into three phenotypes, namely, M2a, M2b, and M2c, and M2c has the strongest suppressive effect [30]. Several studies reported that polarization of the M2c phenotype was induced by IL-10, which is an anti-inflammatory cytokine [30, 31]. In addition, some reports showed that treatment with montelukast increased IL-10 levels in serum and inhibited inflammation [32, 33]. Further experiments are needed to determine how M2 macrophage polarization is influenced by montelukast.

The in vivo experiments in this study investigated Ang II–infused AAA in apoE^−/−^ mice. Angiotensin II infusion promotes macrophage accumulation and a vascular inflammatory response in the adventitia of apoE^−/−^ mice, a mechanism that is similar to that in human AAA models such as those involving calcium chloride or elastase [34]. We showed that orally administered montelukast suppressed the formation and progression of AAA, decreased the degradation of the medial elastin area, regulated the expression of inflammatory proteins in the aortic wall, and inhibited MMP-2 activity. Our findings suggest that montelukast suppresses the destruction of the extracellular matrix in the aortic wall by inhibiting the infiltration of inflammatory factors and attenuating the activity of MMP, resulting in the prevention of AAA formation. Given that montelukast significantly promotes TIMP-2, whose primary role is to regulate MMP-2 enzyme activity, the drug might suppress MMP-2 by affecting TIMP-2. This mechanism differs from that of doxycycline, which is a potent antibiotic against microbial infections and which directly inactivates MMPs by combining with their active zinc site [12, 35]. The finding that montelukast had no significant effect on MMP-9 activity in our in vivo experiment differs from the results of previous studies [11, 27]. In our in vitro experiment, montelukast significantly suppressed MMP-9 gene expression in macrophages. Although MMP-9 is secreted by a large number of cell types, including neutrophils, fibroblasts, and endothelial cells, no studies have investigated whether montelukast influences MMP-9 secretion by each of these cell types. Thus, it is possible that MMP-9 secretion by these cells might have impacted our results.

Limitations of the current study should be mentioned. First, it is important to note that the in vivo dose of montelukast used in this study was higher than the dose generally administered for treatment of asthma. Referring to previous reports [18] and considering that montelukast has few side effects, we selected a dose of 10 mg/kg/day in mice, while in humans the standard dose is 10 mg/day in adults and 5 mg/day in children. Second, although Ang II–infused AAA in apoE^−/−^ mice was used as a model in this study because the underlying pathophysiologic mechanism resembles that of human AAA, we must verify the efficacy of montelukast in another animal model, such as a calcium chloride–induced or elastase-perfusion mouse model.

## 5. Conclusions

Our results suggest that montelukast induces M2 macrophage polarization and prevents AAA formation in apoE^−/−^ mice. Montelukast might have therapeutic potential in preventing the development of AAA.

## Figures and Tables

**Table 1:** Primers used in the quantitative reverse transcription polymerase chain reaction assay.

Gene	Accession No.	Forward and reverse primer (5′-3′)
GAPDH	NM_008084	AACTTTGGCATTGTGGAAGG
		GGATGCAGGGATGATGTTCT

IL-1*β*	NM_008361	CAGGCAGGCAGTATCACTCA
		AGCTCATATGGGTCCGACAG

IL-6	NM_031168	AGTTGCCTTCTTGGGACTGA
		TCCACGATTTCCCAGAGAAC

IL-10	NM_010548	CCAGTTTTACCTGGTAGAAG
		TGTCTAGGTCCTGGAGTCCA

MMP-2	NM_008610	GTCGCCCCTAAAACAGACAA
		GGTCTCGATGGTGTTCTGGT

MMP-9	NM_013599	CGTCGTGATCCCCACTTACT
		AACACACAGGGTTTGCCTTC

TNF-*α*	NM_013693	TATGGCTCTGGGTCCAACTC
		CTCCCTTTGCAGAACTCAGG

MCP-1	NM_011333	CCACTCACCTGCTGCTGCTA
		TGGTGATCCTCTTGTAGCTC

Arginase-1	NM_007482	CAGAAGAATGGAAGAGTCAG
		CAGATATGCAGGGAGTCACC

## Data Availability

All data generated or analyzed during this study are included in this article.

## Supplementary Materials

Supplementary Materials Diagram of our in vivo study protocol. In the montelukast group, mice orally received montelukast (10 mg/kg/day) once a day. The Saline group received the same dosage of normal Saline. Administration of montelukast and Saline were begun immediately after subcutaneous pump implantation. Ultrasonography was performed every week.
