# Supplementary material for: Montelukast, a Cysteinyl Leukotriene Receptor 1 Antagonist, Induces M2 Macrophage Polarization and Inhibits Murine Aortic Aneurysm Formation
Source: Biomed Res Int. 2019 May 27;2019:9104680. doi: 10.1155/2019/9104680 (PMC6556796; doi:10.1155/2019/9104680)
Supplement: Supplementary Materials — Diagram of our in vivo study protocol. In the montelukast group, mice orally received montelukast (10 mg/kg/day) once a day. The Saline group received the same dosage of normal Saline. Administration of montelukast and Saline were begun immediately after subcutaneous pump implantation. Ultrasonography was performed every week. [file 9104680.f1.docx]

**Supplemental figure and figure legends**

Supplemental figure 1


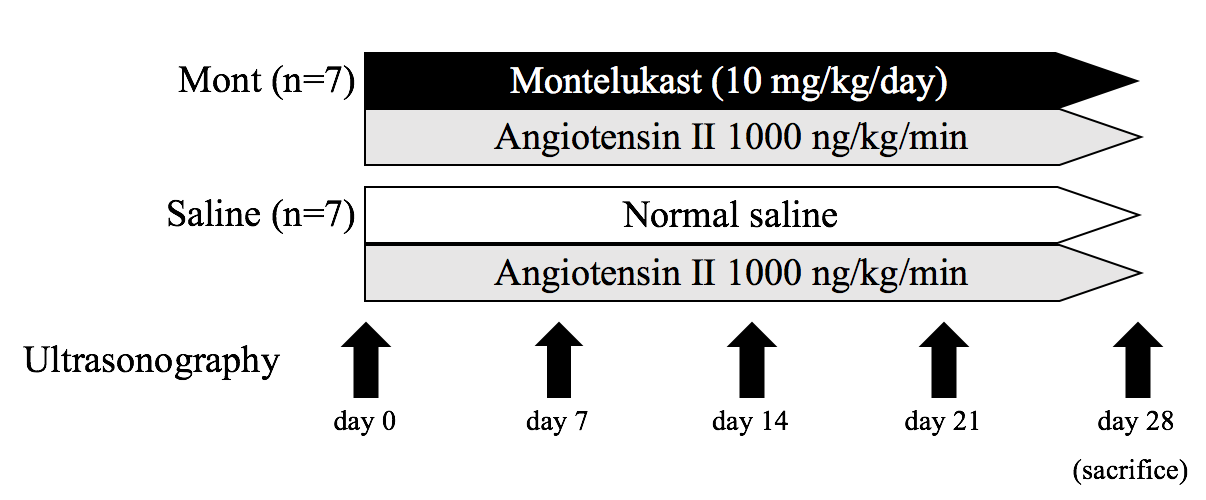


Supplemental figure 1

Diagram of our in vivo study protocol. In the montelukast group, mice orally received montelukast (10 mg/kg/day) once a day. The saline group received the same dosage of normal saline. Administration of montelukast and saline were begun immediately after of subcutaneous pump implantation. Ultrasonography was performed every week.
